# Dasatinib and Quercetin Combination Increased Kidney Damage in Acute Folic Acid-Induced Experimental Nephropathy

**DOI:** 10.3390/ph18060822

**Published:** 2025-05-30

**Authors:** Antonio Battaglia-Vieni, Vanessa Marchant, Lucia Tejedor-Santamaria, Cristina García-Caballero, Elena Flores-Salguero, María Piedad Ruiz-Torres, Sandra Rayego-Mateos, Ana Belen Sanz, Alberto Ortiz, Marta Ruiz-Ortega

**Affiliations:** 1 Molecular and Cellular Biology in Renal and Vascular Pathology Laboratory, Department of Medicine, IIS-Fundación Jiménez Díaz, Universidad Autónoma de Madrid, Avda. Reyes Católicos, 2, 28040 Madrid, Spain; antonio.battaglia@uam.es (A.B.-V.); vanessa.marchant@uam.es (V.M.); lucia.tejedor@quironsalud.es (L.T.-S.); elena.fsalguero@quironsalud.es (E.F.-S.); srayego@fjd.es (S.R.-M.); 2 Ricors2040, Instituto de Salud Carlos III, 28029 Madrid, Spain; asanzb@fjd.es (A.B.S.); aortiz@fjd.es (A.O.); 3 Inflammation and Immunopathology of Organs and Systems Laboratory, University Hospital La Princesa, C/Diego de León 62, 28006 Madrid, Spain; crisgcomplutense@gmail.com; 4 Department of Systems Biology, University of Alcalá, 28871 Alcalá de Henares, Spain; mpiedad.ruiz@uah.es; 5 Division of Nephrology and Hypertension, IIS-Fundación Jiménez Díaz, Universidad Autónoma Madrid, 28040 Madrid, Spain

**Keywords:** acute kidney injury, senolytics, senescence, dasatinib, quercetin, Klotho, p21

## Abstract

**Background/Objectives**: Acute kidney injury (AKI) remains an unsolved medical problem due to the lack of effective treatments, high mortality, and increased susceptibility to progression to chronic kidney disease (CKD), especially in the elderly. Cellular senescence has been described in AKI, CKD, and aging and has been proposed as a promising therapeutic target. The senolytic drug combination of dasatinib plus quercetin (D&Q) is beneficial in some pathological conditions, including experimental CKD, but there are no data for AKI. **Methods**: The effect of D&Q combination was tested in folic acid-induced nephrotoxicity (FAN-AKI), a murine AKI model. **Results**: D&Q pretreatment did not prevent renal dysfunction in the acute phase of FAN-AKI, as determined by serum creatinine and BUN levels at 48 h. Moreover, gene expression of the kidney damage biomarkers *Lcn2* and *Havcr1*, the *Cdkn1a* gene, which encodes p21, and some genes encoding components of the senescent cell secretome were significantly increased in response to D&Q treatment. The number of senescent p21-positive cells in injured kidneys was similar in untreated or D&Q-treated FAN mice. In addition, D&Q did not prevent the downregulation of the antiaging factor *Klotho* in damaged kidneys. **Conclusions**: D&Q treatment was not protective in FAN-AKI, exacerbating some deleterious responses. These results suggest caution when exploring the clinical translation of D&Q senolytic activity.

## 1. Introduction

Acute kidney injury (AKI) is the most common cause of death in hospitalized and clinically ill patients [1,2,3]. It is characterized by a sudden but often reversible reduction in renal function, resulting in changes in serum creatinine and urine levels that may progress to chronic kidney disease (CKD) [2,4]. Although, in 2013, the International Society of Nephrology launched an initiative to eradicate or, at least, reduce preventable AKI-related deaths worldwide by 2025 [5], this goal is far from being achieved. To date, no pharmacological strategies have been implemented to prevent nor to cure AKI episodes, and renal replacement therapies remain the standard of care [6]. Population aging is contributing to a higher burden of both AKI and CKD. By 2040, it is estimated that CKD will become the fifth global leading cause of death [7]. Both CKD and older age increase the risk of AKI and of the AKI-to-CKD progression [8]. This highlights the importance of further research to find interventions that prevent or alleviate AKI.

Activation of cellular senescence has been described both in AKI and CKD [9]. Cellular senescence occurs in both physiological (e.g., embryonic development, tissue regeneration and repair) and pathological (e.g., metabolic disorders, aging and age-related diseases) processes [10,11] and in response to multiple stressors (e.g., DNA damage, epigenetic changes, metabolic shocks, oxidative stress). Cellular senescence is characterized by increased expression of cell-cycle-related molecules, such as the cyclin kinase inhibitors (CDK) p16ink4a (p16; encoded by *CDKN2A*), and p21WAF-1/CAP1 (p21, encoded by *CDKN1A*), that initiate and maintain a stable cell cycle arrest in the G1 or possibly G2 phase, promoting resistance to apoptosis [12]. Phenotype changes in senescent cells include an aberrant secretome, namely the “senescence-associated secretory phenotype” (SASP), containing proinflammatory cytokines, growth factors, and chemokines [13]. Renal accumulation of senescent cells has been described in CKD patients, and these cells can contribute to kidney damage progression through the release of SASP proinflammatory and profibrotic factors [14]. However, the role of cellular senescence in the early phase of AKI is not fully understood [15,16]. The acute phase of injury is followed by regeneration consisting of the proliferation of tubular cells and restoration of epithelial phenotype and function [17]. Some cells do not recover and are thought to acquire a senescent phenotype, leading to maladaptive repair promoting the AKI-to-CKD transition [15,18]. In this sense, targeting senescence in maladaptive repair, renal fibrosis, and transplant failure was beneficial in preclinical models [19,20].

Senotherapeutic strategies to target senescence are divided into senolytics, which eliminate senescent cells, and senomorphics, which suppress the pathological SASP [21]. Senolytics may target prosurvival pathways, including the BCL-2/BCL-xL, p53/p21, and PI3K/AKT pathways, and antiapoptotic pathways, including serpins [22]. The senolytic combination of dasatinib and quercetin (D&Q) was discovered by a hypothesis-driven bioinformatics approach, and this combination has demonstrated senolysis in vitro and in vivo [23]. Dasatinib is a tyrosine inhibitor of BCR-ABL, SRC family, c-KIT, and ephrin receptors, used to treat leukemias, that enhances apoptosis and inhibits proliferation and migration [21]. Quercetin is a flavonoid inhibitor of PI3Ks and serpins, with anti-inflammatory, antioxidant and antiproliferative properties [24]. In a model of type I-diabetes contrast-induced AKI, quercetin demonstrated a dose-dependent (25 to 75 mg/kg) renal protective action by the activation of the Sirt1 pathway targeting oxidative deleterious effects [25]. While both compounds have individual proapoptotic properties, the D&Q combination exerts an apoptotic effect that has made it a valuable tool in senescence-targeting studies [25,26]. In experimental diabetic nephropathy, the administration of the D&Q combination for 10 weeks improved renal function [26], as described in other models of CKD [20]. However, preclinical data on the safety and efficacy of D&Q in the acute phase of AKI are unknown. Therefore, our aim was to investigate the effect of the senolytic drug cocktail D&Q in experimental AKI caused by folic acid nephrotoxicity (FAN-AKI).

## 2. Results

### 2.1. Combination of Dasatinib Plus Quercetin Did Not Prevent Renal Dysfunction in FAN-AKI

**Figure 1:**
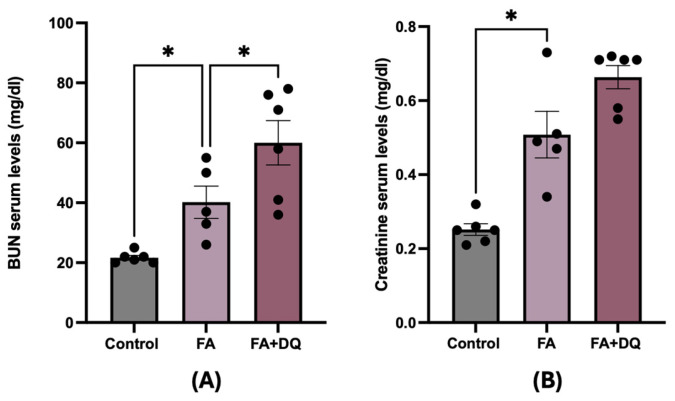
Dasatinib plus quercetin (D&Q) increased renal dysfunction in FAN-AKI. Mice were injected with folic acid (125 mg/kg IP) and sacrificed 48 h later. Two groups were studied: one received a single oral dose of the combination of D&Q (dasatinib, 5 mg/kg body mass and quercetin, 50 mg/kg body mass, via oral gavage), and another group remained untreated. (**A**) BUN and (**B**) Creatinine were evaluated in serum. Data are expressed as the mean of 5–6 animals per group ± SEM. * *p* < 0.05. One-way ANOVA was followed by the Holm–Šídák multiple comparison test for BUN, while for creatinine, the Kruskal–Wallis non-parametric statistical test was followed by Dunn’s test without correction.

To investigate the effect of D&Q in AKI, nephrotoxicity was induced in C57BL/6 mice by an overdose of folic acid, and mice were studied at 48 h. FAN is an established model to investigate the molecular mechanism involved in the acute phase of AKI [27]. Serum BUN and creatinine were increased in FAN-AKI at 48 h compared to controls (Figure 1A,B), indicating decreased kidney function in the acute phase of AKI. However, D&Q pretreatment did not improve serum renal functionality markers compared to untreated FAN-AKI mice (Figure 1A,B), suggesting that this senolytic pretreatment does not protect from the acute phase of AKI.

### 2.2. Dasatinib Plus Quercetin Increased the Gene Expression of Kidney Damage Biomarkers in FAN-AKI

**Figure 2:**
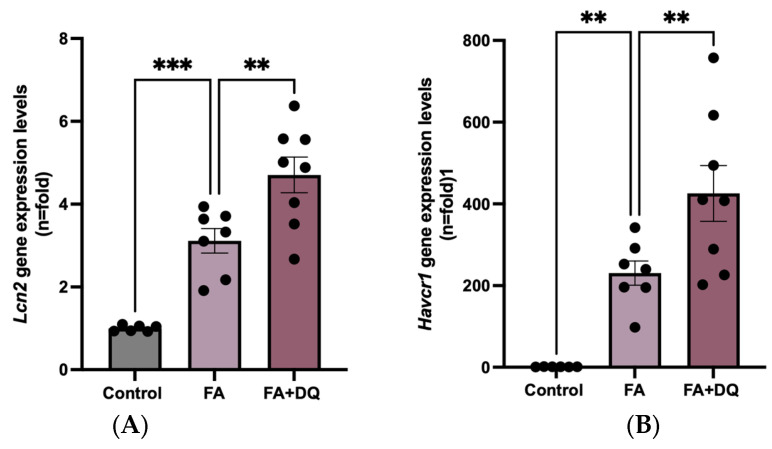
Dasatinib plus quercetin (D&Q) increased the gene expression of kidney injury biomarkers in FAN-AKI. RNA was extracted from total kidney cortex, and Lcn2 and Havcr1 gene expression (**A**,**B**) was evaluated by real time PCR. Data are expressed as the mean of 6–8 animals per group ± SEM. ** *p* < 0.005, *** *p* < 0.0005. One-way ANOVA was followed by the Holm–Šídák multiple comparison test.

To further evaluate the effect of D&Q on FAN-AKI, the gene expression of well-known kidney damage biomarkers *Havcr1* and *Lipocalin-2* (*Lcn2*), encoding the proteins KIM-1 and NGAL, respectively [28], was studied. In FAN-AKI, qPCR disclosed increased kidney *Lcn2* and *Havcr1* gene expression (Figure 2A,B) compared to healthy controls, as previously described in the acute phase of AKI [29]. In response to D&Q pretreatment, the gene expression of both biomarkers significantly increased in injured FAN-AKI kidneys compared to untreated FAN-AKI (Figure 2A,B), suggesting a deleterious effect and an increased severity of kidney injury resulting from senolytic pretreatment. 

### 2.3. Dasatinib and Quercetin Did Not Modify the Tubular Damage Marker KIM-1 in FAN-AKI

**Figure 3:**
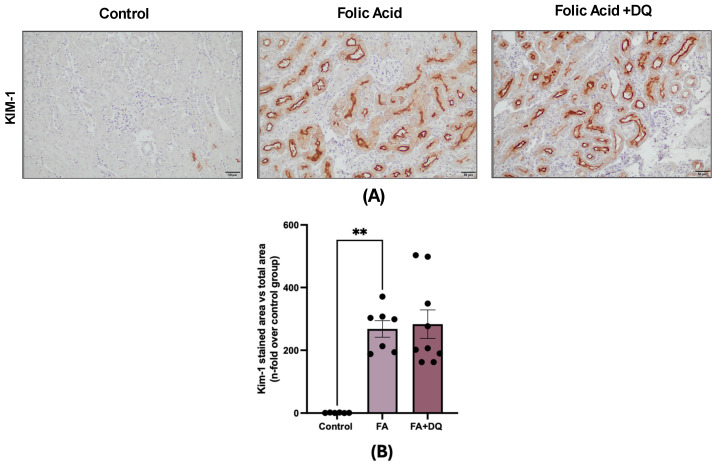
Dasatinib + quercetin (D&Q) did not modify the tubular damage marker KIM-1 in FAN-AKI. (**A**) Representative microphotographs of KIM-1 immunohistochemistry at 20× magnification. (**B**) Quantification of KIM-1 immunohistochemical staining expressed as mean stained area relative to the total area, represented as n-fold. Data are expressed as mean of 6 to 9 animals per group ± SEM. ** *p* < 0.005. The Kruskal–Wallis non-parametric statistical test was followed by Dunn’s test without correction.

During FAN-AKI, injured tubular cells de novo express KIM-1, a marker of tubular damage (Figure 3A,B). The number of KIM-1-positive cells was similar between untreated and D&Q-treated FAN-AKI (Figure 3B). 

### 2.4. Dasatinib Plus Quercetin Did Not Modify the Number of Senescent Cells in FAN-AKI

**Figure 4:**
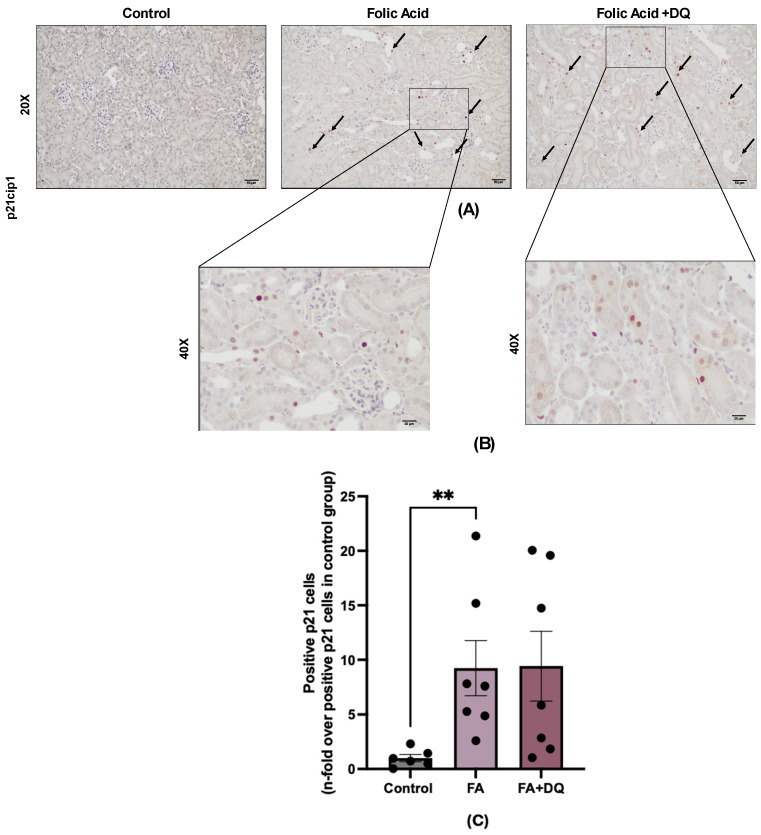
Dasatinib + quercetin (D&Q) did not modify the number of senescent cells in FAN-AKI. (**A**) Representative microphotograph of p21 immunohistochemistry at 20× magnification. (**B**) Representative microphotograph of p21 immunohistochemistry at 40× magnification. (**C**) Quantification of p21-positive cells in eight fields for each sample, represented as n-fold. The arrows indicate the positive p21 cells. Data are expressed as the mean of 6 to 7 animals per group ± SEM. ** *p* < 0.005. The Kruskal–Wallis non-parametric statistical test was followed by Dunn’s test without correction.

To test the potential senolytic effect of D&Q in FAN-AKI, we evaluated its impact on the number of tubular senescent cells. To this aim, cells stained for p21, a universal marker of cell senescence, were evaluated by immunohistochemistry. In FAN-AKI, p21-positive cells were observed mainly among tubular cells, whereas no staining was found in healthy controls (Figure 4A). D&Q did not modify the number of p21-positive cells compared to untreated FAN-AKI mice (Figure 4B). These results suggest the lack of a senolytic effect of D&Q in the acute phase of FAN-AKI.

### 2.5. Dasatinib Plus Quercetin and Senescence-Associated Biomarkers

**Figure 5:**
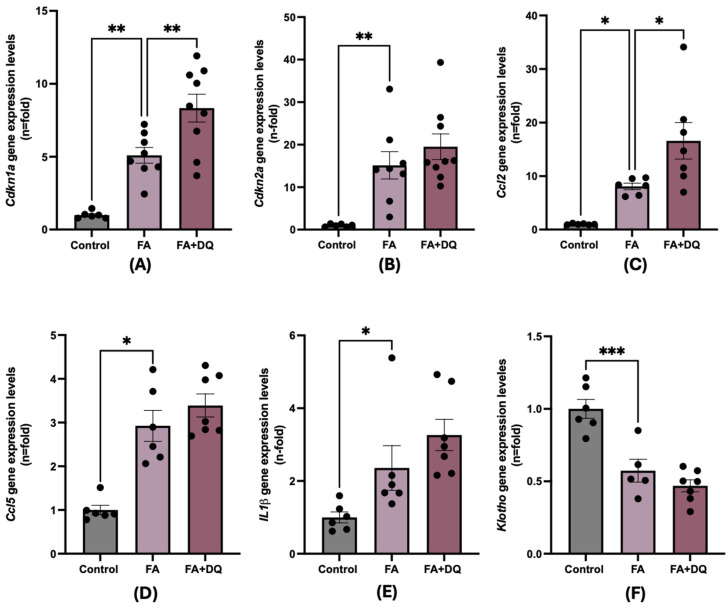
Dasatinib + quercetin (D&Q) did not decrease senescence-associated markers in FAN-AKI. The D&Q pretreatment effect on gene expression was evaluated by RT qPCR. (**A**) Cdkn1a; (**B**) Cdkn2a; the SASP components (**C**) Ccl2; (**D**) Ccl5; (**E**) IL1β; and (**F**) the antiaging factor Klotho. Data are expressed as the mean of 6 to 7 animals per group ± SEM. * *p* < 0.05 ** *p* < 0.005 *** *p* < 0.0005. One-way ANOVA was followed by the Holm–Šídák multiple comparison test, except for Ccl5, where the Kruskal–Wallis non-parametric statistical test was followed by Dunn’s test without correction.

To test whether D&Q modulated other senescence-associated processes in FAN-AKI, we analyzed the gene expression of well-established markers of cell senescence in mouse kidneys. First, the expression of the cell cycle arrest genes *Cdkn1a* and *Cdkn2a*, that encode p21 and p16, respectively, was evaluated. In FAN-AKI, *Cdkn1a* and *Cdkn2a* expression increased compared with healthy control (Figure 5A,B), as previously described [30]. However, in D&Q-treated mice, renal *Cdkn1a* gene expression was significantly increased compared to untreated FAN-AKI mice (Figure 5A,B).

Senescent cells are characterized by gene reprograming, leading to increased SAPS gene expression [31]. The gene expression for several SASP components, such as the proinflammatory cytokine *IL1β* and the chemokines *Ccl2* and *Ccl5*, was significantly higher in FAN-AKI compared to controls, as described [30,32]. However, D&Q did not diminish SASP proinflammatory gene expression, whereas it significantly increased *Ccl2* expression (Figure 5D,E). These data indicate an absence of an anti-inflammatory effect of D&Q in FAN-AKI.

The antiaging factor *klotho* is downregulated in response to kidney damage [32]. In FAN-AKI, *klotho* mRNA levels decreased and were not restored by D&Q (Figure 5).

### 2.6. Dasatinib Plus Quercetin Did Not Neither Increase Apoptosis nor Modify Necroptosis Pathway Activation During FAN-AKI

**Figure 6:**
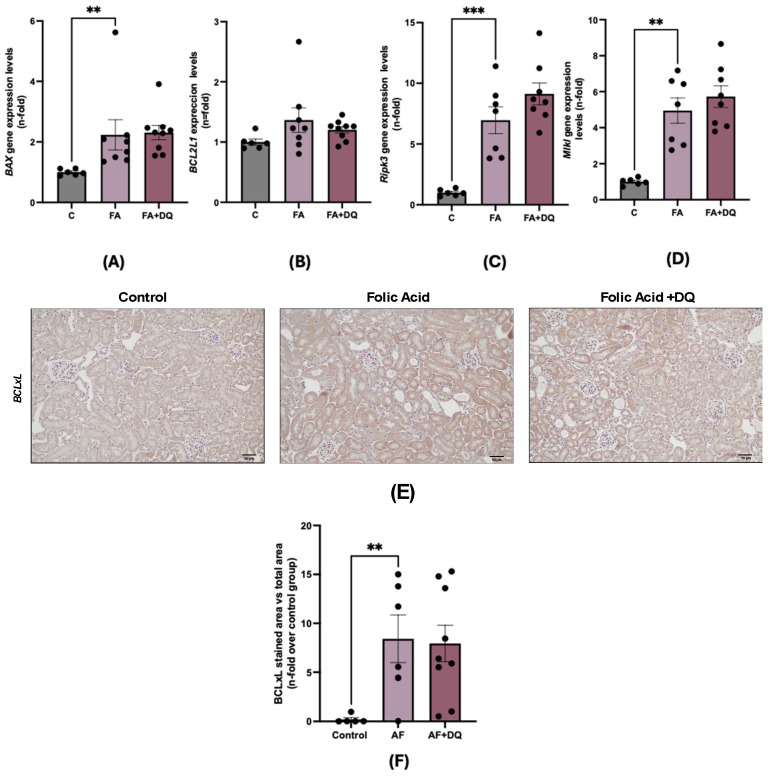
Dasatinib + quercetin (D&Q) did not increase apoptosis or necroptosis in FAN-AKI. The impact of D&Q pretreatment on apoptosis was evaluated both at the gene (RT qPCR) and protein levels. (**A**) BAX; (**B**) BCL2L1; (**C**) *Ripk3*; (**D**) *Mlkl* renal gene expression. (**E**) Representative microphotographs of positive BCLxL per field at 20× magnification. (**F**) Quantification of BCLxL immunohistochemical staining expressed as mean stained area relative to the total area, represented as n-fold. Data are expressed as mean of 4 to 9 animals per group ± SEM. ** *p* < 0.005, *** *p* < 0.0005. The Kruskal–Wallis non-parametric statistical test followed by Dunn’s test without correction was performed for apoptosis markers, while for necroptosis ones, one-way ANOVA followed by the Holm–Šídák multiple comparison test were carried out.

D&Q targets prosurvival and antiapoptotic pathways [22]. To assess the proapoptotic activity of D&Q in FAN-AKI, the expression of apoptotic and antiapoptotic genes (*Bax* and *Bcl2l1*, respectively) was evaluated. In FAN-AKI, *Bax* expression increased compared to untreated kidneys, and was not influenced by D&Q (Figure 6A). Moreover, the antiapoptotic factor *Bcl2l1* (which encodes BCLxL) was evaluated at both gene and protein levels. No significant changes were observed for gene expression (Figure 6B), whereas for BclxL, protein increased in FAN-AKI when compared to control, but this was not modified by D&Q (Figure 6F). Moreover, previous studies have described the cell death mechanisms involved in experimental FAN-AKI [27,32], emphasizing the role of necroptosis, including the upregulation of key components in this cell death pathway, such as receptor-interacting protein kinase-3 (RIPK3) and mixed-lineage kinase domain-like protein (MLKL) [32]. Pretreatment with D&Q had no effect on the *Ripk3* and *Mlkl* gene overexpression observed in FAN-AKI (Figure 6C,D).

### 2.7. Gender Differences Were Observed in Response to Folic Acid-Induced Renal Injury, Whereas Similar Deleterious Senolytic Effect Was Found in Male and Female Mice

**Figure 7:**
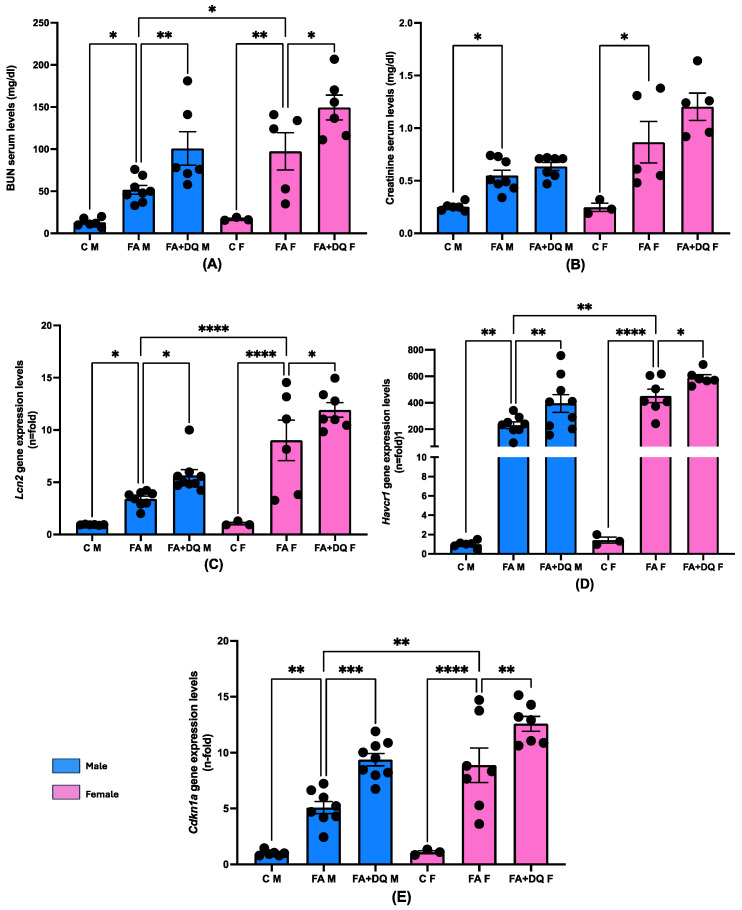
No gender-related responses to dasatinib + quercetin (D&Q) therapy in FAN-AKI. The impact of D&Q pretreatment was further evaluated in female mice, testing several damage markers. (**A**) BUN and (**B**) creatinine were determined in serum. In the kidney, the damage biomarkers (**C**) *Lcn2*, (**D**) *Havcr1*, and the senescent biomarker (**E**) *Cdkn1a* were assessed at gene level. Data are expressed as mean of 4 to 9 animals per group ± SEM. * *p* < 0.05, ** *p* < 0.005, *** *p* < 0.0005, **** *p* < 0.00005. The Kruskal–Wallis non-parametric statistical test followed by Dunn’s test without correction was performed for apoptosis markers, while for necroptosis ones, one-way ANOVA followed by the Holm–Šídák multiple comparison test were performed. Female mice are represented by pink bars, while male mice are represented by blue ones. Please note that the data for male mice are identical to the previous figures in this manuscript. This was included to facilitate a more accurate gender comparison.

Although analyzing gender differences was not the main focus of our study, we compared the nephrotoxic effects of FA and the D&Q combination treatment in male and female mice. In response to FA administration, females presented increased levels of serum (BUN) and renal damage markers (Havcr1 and Lcn1) and higher levels of Cdkn1a (Figure 7) at 48 h when compared to males (Figure 7). The senolytic combination pretreatment affects both genders similarly, worsening the damage in female and male mice (Figure 7).

## 3. Discussion

The main finding is that a senolytic drug combination, D&Q, can increase renal damage in the acute phase of experimental nephrotoxic nephropathy. Clinical trials are evaluating the senolytic potential of D&Q in multiple conditions, including Alzheimer’s disease (NCT05422885), skeleton health (NCT04313634), pulmonary fibrosis (NCT02874989), and diabetic CKD (NCT02848131), but none have reported on kidney function. To our knowledge, there are no studies evaluating the effect of D&Q in the acute phase of experimental models of AKI, nor evaluating the treatment’s long-term kidney safety, emphasizing the need to report negative results of drugs already in clinical development in humans, especially in drug repurposing.

Genetic or pharmacological elimination of senescent cells was beneficial in many murine preclinical models of disease [22]. Therefore, targeting senescent cells in aging and age-related diseases has been proposed as a potential therapeutic option for humans. The beneficial effects of D&Q were initially described in old mice [33,34,35] observing a longer lifespan [35]. In an open-label phase 1 clinical trial (NCT02848131), treatment with D&Q for 3 days decreased adipose tissue senescent cell burden in diabetic patients [36]. In diabetic db/db mice, treatment with D&Q for 20 weeks reduced glycemia and improved functional and histopathological changes [37]. D&Q also attenuated adipose tissue inflammation and improved systemic metabolic function in old mice without changing senescence and inflammatory SASP markers in the liver and skeletal muscle [33]. In line with these data, D&Q treatment did not diminish senescence and inflammatory SASP markers in injured FAN-AKI kidneys. These data suggest that the beneficial D&Q effects described in diabetes can be restricted to senolysis on adipose tissue or other specific tissues. In this sense, in a murine model of 5/6 nephrectomy that resembles human nephron reduction in CKD, D&Q treatment reduced muscle wasting, suggesting that skeletal muscle can also be a target tissue [38]. However, they did not report kidney function, damage, or senescence parameters. In muscle wasting, idiopathic pulmonary fibrosis, and intestinal senescence, D&Q decreased senescence markers (p21, p16) and SASP components [34,38,39]. By contrast, D&Q reduced the senescent-like myeloid cells in experimental autoimmune encephalomyelitis but did not influence inflammation or mice recovery [40]. Other senolytics, such as navitoclax (ABT-263) and fisetin, were beneficial in cardiovascular disease [41]. However, the role of senolytics in renal regeneration has not been fully elucidated.

Proximal tubule cells are the kidney cells most sensitive to nephrotoxins [42]. Nephrotoxic insults elicit different stress responses in injured tubular cells [17], including activation of CDK inhibitors, mainly p21 [43]. In different experimental AKI models, including ischemia-reperfusion and cisplatin nephrotoxicity, rapid activation of cell senescence was observed; this was mainly induction of p21 gene and protein expression in tubular cells [44,45,46,47,48], as observed for FAN-AKI. Early p21 activation has been interpreted as a protective mechanism, preventing uncontrolled progression to cell death, allowing damaged cells to remain in cell cycle arrest, and thus providing more time for DNA damage repair [46]. In addition, p21 activation modulates apoptosis and necrosis [49]. Indeed, AKI was more severe in p21 knockout mice [49]. D&Q significantly increased *p21* gene expression in FAN-AKI, and it did not diminish the number of p21-positive tubular senescent cells. A possible explanation for the loss of the senolytic effect could be the overall net balance between apoptosis and senescence in this acute phase of AKI characterized by cell death (necroptosis). Accordingly, D&Q did not modify AKI-induced changes on the antiapoptotic protein BCLxL or on necroptosis-related components. Some data suggest that activation of cellular senescence can be protective in the early stages of AKI [15], indicating that D&Q could be deleterious in the acute phase of AKI, characterized by ongoing cell death. Supporting this finding, biomarkers of renal damage *Havcr1* and *Lcn2*, and the proinflammatory mediator *ccl2* genes, were significantly overexpressed in response to D&Q treatment in mice with FAN-AKI.

Contrary to our findings, in preclinical septic AKI induced by LPS administration, the senolytic compound fisetin [50] inhibited kidney dysfunction, inflammation, and apoptosis [51]. Fisetin is a flavonoid that diminished renal inflammation in many preclinical models [52,53,54,55,56], therefore exerting senomorphic activities by inhibiting SAPS overexpression. Moreover, anti-inflammatory treatments ameliorate LPS-induced AKI [57]. In this sense, fisetin’s inhibition of the TLR4-NF-κB p65 and MAPKs pathways in LPS-AKI [51] may explain or contribute to its beneficial effects, independent of potential senolytic actions. There is ample evidence of the beneficial effects of SASP inhibitors in preclinical studies, such as those observed for the IL-1β receptor inhibitor anakinra, used in rheumatoid arthritis, or metformin, a treatment for type 2 diabetes which inhibits the transcription factor NF-κB [31]. Reports also support the beneficial effect of SASP-targeting senomorphic therapies in the acute phase of AKI. Blockade of the SASP component CCN2 improved the acute phase of experimental IRI-AKI and FAN-AKI by targeting oxidative stress [58] and the NLRP3/RIPK3/NRF2 pathway [59]. IKK/NF-κB and JAK inhibitors also suppress the SASP [60]; however, no protective effect has been described in acute AKI.

In contrast to AKI findings, there are reports of protective effects of senolytics in experimental CKD. Suppression of tubular senescence by navitoclax and fisetin attenuated renal fibrosis and improved tubular repair, as indicated by the restoration of tubular regeneration and renal function [15,18]. Navitoclax inhibits Bcl-2 family members, thereby activating apoptosis mechanisms in senescent cells. In murine cisplatin AKI, navitoclax inhibited tubular senescence, improving fibrosis and renal function [18]. However, navitoclax did not exhibit antisenescence properties in premature aging in short telomere zebrafish [61]. Navitoclax may produce thrombocytopenia as a dose-limiting adverse effect [21].

Klotho is a protein mainly synthetized by the kidneys [62] and downregulated in AKI and CKD [63]. The administration of recombinant Klotho protein in experimental CKD delayed fibrosis [64]. Therefore, therapies restoring Klotho were proposed as nephroprotective and antiaging agents [65]. Exposure to the senescent cell secretome reduces α-Klotho in different cultured human cells, and this was partially prevented by blocking some SASP factors such as IL1α [65]. In an idiopathic pulmonary fibrosis clinical trial (NCT02874989), D&Q improved physical function and increased urinary α-Klotho [65], suggesting that D&Q could modulate Klotho levels. However, the mechanisms involved are poorly understood. By contrast, in FAN-AKI, renal *Klotho* downregulation was not restored by D&Q treatment [66].

Aging is a complex process [11]. Senescent cell burden increases in multiple tissues with aging [67]. Cellular senescence may accelerate aging, as the transplantation of a small number of senescent cells in young mice led to physical dysfunction [35]. Moreover, genetic or pharmacological elimination of senescent cells extended the health span and longevity of naturally aged mice [22,35], as well as in mice with accelerated aging [23]. In humans and in preclinical AKI, aging increased the severity of renal injury [8]. The acute phase of FAN-AKI was more severe in aged mice, as evidenced by higher tubular cell death (mainly regulated necrosis), suppressed apoptosis, a higher number of senescent cells, and increased levels of senescent markers (p16, p21, γH2AX, SASP components) [30]. These data suggest that overactivation of senescence mechanisms, including suppression of apoptosis, could contribute to increasing the severity of AKI in the elderly. In this regard, FOXO4-DRI, a senolytic drug targeting the p53-FOX4 interaction that promotes apoptosis of senescent cells, improved kidney function in mice with spontaneous or accelerated aging [68]. However, strategies that eliminate senescent cells could be detrimental in certain disease conditions, as described here for FAN-AKI in the first study to evaluate D&Q in an AKI model. Our results indicate that targeting senescence in AKI may require a more tailored approach, as the benefits observed in other contexts, such as aging or chronic diseases, do not necessarily translate to AKI. Although this study does not support the use of D&Q in AKI, further research is essential to better understand the molecular mechanisms triggered by senolytic drugs in different contexts.

Certain limitations should be acknowledged. Although FAN-AKI shares some cellular and molecular mechanisms with other causes of AKI, future studies should address other causes of AKI, including IRI and sepsis-induced AKI. In addition, D&Q was tested at a single dose and timepoint, in a preventive manner. Whether this is the optimal therapeutic time window and dose remain to be determined. In addition, the effect of single or repeated D&Q administration or its vehicle in the kidney of healthy mice has not been tested. This study only evaluated D&Q effects in young mice, whereas age-related effects will require further investigation. In this sense, D&Q worsened obesity and age-dependent experimental liver disease progression has been reported [69]. Finally, this preclinical study requires clinical confirmation that may be derived from the evaluation of renal safety in ongoing clinical trials. 

In conclusion, in preclinical nephrotoxic AKI, preventive D&Q treatment was not protective and even increased the severity of some features of kidney damage in the acute phase. This is a cautionary tale for the clinical translation of senolytic therapies.

## 4. Materials and Methods

### 4.1. Animals

Experiments were performed according to the European Community guidelines for animal experiments and the ARRIVE guidelines and with consent of the Experimental Animal Ethics Committee of the Health Research of the IIS-Fundación Jiménez Díaz and PROEX 242.2/21 (29 April 2021) and PROEX 065/18 of the Community of Madrid. Animals were sacrificed with an overdose of CO_2_ in a special chamber. Blood and urine were collected, and kidneys were perfused in situ with saline before removal. Half of each kidney (2/4) was fixed, embedded in paraffin, and used for immunohistochemistry, and the rest was snap-frozen in liquid nitrogen for renal cortex RNA studies.

### 4.2. Folic Acid Model (FAN-AKI)

AKI was induced in male and female C57BL/6 mice of 3 months of age by a single intraperitoneal (i.p.) injection of 125 mg/kg body weight folic acid (Sigma-Aldrich, St. Louis, MO, USA) in 0.3 mol/L sodium bicarbonate. Animals were sacrificed 48 h later. Experimental FA-AKI recapitulates the sequence of pathological, as well as adaptive responses, during human AKI, including several cell death programs involved in apoptosis and regulated necrosis [32,70]. A total of 24 h before, some animals received folic acid, others received a single oral dose of the combination of dasatinib (5 mg/kg body weight) and quercetin (50 mg/kg body weight) in polyethylene glycol 10% (Sigma-Aldrich), and others remained untreated (*n* = 6 to 9 mice per group). The dosage and administration schedule of the D&Q senolytic combination was selected based the pharmacokinetics of each drug [71,72], previously reported beneficial effect of D&Q senolytic combination at the same dose every 3 days [26,38], as well as pretreatment therapies in FAN-AKI [70,73]. In pilot studies, no adverse renal effects were detected with vehicle (polyethylene glycol 10% or 0.3 mol/L sodium bicarbonate).

### 4.3. Biochemical Studies

Serum BUN and creatinine were measured at IIS-FJD using a Cobas C702 module (Roche Diagnostics, Rotkreuz, Switzerland), which utilizes photometric detection methods. Creatinine and urea quantification was performed according to the manufacturer’s instructions for the enzymatic Creatinine Plus Version 2 reagent assay (Roche Diagnostics, Rotkreuz, Switzerland) and UREAL assay (Roche Diagnostics, Rotkreuz, Switzerland).

### 4.4. Gene Expression Studies

RNA from renal cortex was isolated with TRItidy G^TM^ (PanReac; Barcelona, Spain). cDNA was synthesized by a High Capacity cDNA Archive kit (Applied Biosystems, Waltham, MA, USA) using 2 μg of total RNA and following the manufacturer’s instructions. Quantitative gene expression analysis was performed on a QuantStudio™ 3 fast real-time PCR system (Applied Biosystems, Waltham, MA, USA) using fluorogenic TaqMan MGB probes and primers designed by Assay-on-Demand^TM^ gene expression products or Predesigned qPCR. Mouse assay IDs were as follows. *Ccl2*: Mm00441242_m1, *Ccl5*: Mm01302428_m1, *Cdkn1a*: Mm00432448_m, *Cdkn2a*: Mm00494449_m1, *Ripk3*: Mm00444947_m1 Havcr1: Mm00506686_m1, *Il1β*: Mm00434228_m1, *Mlkl*: Mm01244219_m1, *Klotho*: Mm00502002_m1, and *Lcn2*: Mm01324470_m1. Data were normalized to *Gapdh*: Mm99999915_g1. The mRNA copy numbers were calculated for each sample by the instrument software using Ct value (“arithmetic fit point analysis for the lightcycler”). Results were expressed in n-fold, calculated relative to control group after normalization against *Gapdh*.

### 4.5. Immunohistochemistry

Paraffin-embedded kidney sections were stained using standard histology procedures, as described elsewhere [74]. Immunohistochemistry (IH) was performed in 3 μm thick tissue sections. Antigens were retrieved using the PTlink system (DAKO) with sodium citrate buffer (10 mM) adjusted to pH 6–9, depending on the immunohistochemical marker. Endogenous peroxidase was blocked. Sections were incubated for 1 h at room temperature with Casein Solution (Vector Laboratories, Newark, CA, USA) to remove non-specific protein binding sites. Then, primary antibodies were incubated overnight at 4 °C. Specific biotinylated secondary antibodies (Amersham Biosciences, Amersham, UK) were used. The latter was followed by Avidin-Biotin Complex incubation (Vector Laboratories, Newark, CA, USA). Signal was developed with substrate solution and 3,3-diaminobenzidine as a chromogen (Abcam, Cambridge, UK). Finally, slides were counterstained with Carazzi’s haematoxylin (Richard Allan Scientific, San Diego, CA, USA).

The primary antibodies used were [dilution] P21cip1 ([1:200, Ab188224, Abcam, Cambridge, UK), BCL-xL ([1:2000], ab178844, Abcam, Cambridge, UK), and KIM-1 ([1:200]; AF 1817, R&D).

Specificity was checked by omission of primary antibodies. Quantification was carried out by using the Image-Pro Plus software version 7.1 (Media Cybernetics, Rockville, MD, USA) to determine the positive staining area relative to the total area or counting positive staining manually (in the case of P21cip1 immunohistochemistry), in 5–10 randomly chosen fields (×200 magnification).

### 4.6. Statistical Analysis

Kidneys from all groups were compared to control and to FAN-AKI, expressing results as fold-change over control values of 1. Results are expressed as fold increase with respect to the control average as mean ± standard error of the mean (SEM) of 4 to 9 animals per group. The Shapiro–Wilk test was used to evaluate sample normality distribution. When samples followed the Gaussian distribution, a one-way ANOVA followed by the corresponding post hoc analyses were used. To compare non-parametric samples, a Kruskal–Wallis and a subsequent post hoc analysis were performed. Graphics and statistical analysis were conducted using GraphPad Prism 9.5.1 (GrahPad Software, San Diego, CA, USA). Values of *p* < 0.05 were considered statistically significant.

## Data Availability

The original contributions presented in this study are included in the article. Further inquiries can be directed to the corresponding author.

## Abbreviations

The following abbreviations are used in this manuscript:

AKI	Acute kidney injury
CKD	Chronic kidney disease
D&Q	Dasatinib (D) and quercetin (Q)
SASP	Senescence-associated secretory phenotype
